# Complete remission of paraneoplastic vanishing bile duct syndrome after the successful treatment of Hodgkin’s lymphoma: a case report and review of the literature

**DOI:** 10.1186/1756-0500-7-529

**Published:** 2014-08-14

**Authors:** Delia Rota Scalabrini, Daniela Caravelli, Fabrizio Carnevale Schianca, Lorenzo D’Ambrosio, Francesco Tolomeo, Paola Boccone, Antonio Manca, Giovanni De Rosa, Annamaria Nuzzo, Massimo Aglietta, Giovanni Grignani

**Affiliations:** Division of Oncology, Candiolo Cancer Institute, FPO, IRCCS, University of Torino Medical School, Candiolo, Italy; Radiology Unit, Candiolo Cancer Institute, FPO, IRCCS, Candiolo, Italy; Division of Surgical Pathology, Ospedale Umberto I Ordine Mauriziano, Torino, Italy

**Keywords:** Vanishing bile duct syndrome, Ductopenia, Hodgkin’s lymphoma, Ursodeoxycholic acid, Paraneoplastic syndrome

## Abstract

**Background:**

Vanishing bile duct syndrome has been associated with different pathologic conditions (adverse drug reactions, autoimmune diseases, graft versus host disease, and cancer). Though its causes are unknown, an immune-related pathogenesis is the most likely one. Vanishing bile duct syndrome can evolve to hepatic failure and, eventually, to death. The treatment is uncertain, but it needs the resolution of the underlying pathologic condition.

**Case presentation:**

We describe the association of Hodgkin’s lymphoma with a syndrome characterized by cholestasis, aminotransferase elevation and an histological picture of bile duct loss. All other causes of hepatic function impairment were excluded (in particular, drugs, viral and autoimmune related diseases) eventually leading to the diagnosis of vanishing bile duct syndrome. Despite the fact that the dysfunction is not caused by hepatic Hodgkin’s lymphoma involvement, liver impairment can limit the optimal therapy of Hodgkin’s lymphoma. A treatment consisting of ursodeoxycholic acid, prednisone, and full dose chemotherapy restored hepatic function and achieved complete and long-lasting remission of Hodgkin’s lymphoma.

**Conclusion:**

We reviewed all case reports showing that vanishing bile duct syndrome is a dismal paraneoplastic syndrome being fatal in a high proportion of patients if not adequately treated. Indeed, this syndrome requires both an early recognition and an appropriate aggressive treatment consisting of full dose upfront chemotherapy which is the only way to achieve a resolution of the vanishing bile duct syndrome. Delayed or reduced intensity treatments unfavorably correlate with survival.

## Background

Vanishing bile duct syndrome (VBDS) is defined as an intrahepatic cholestasis with paucity of interlobular bile ducts [1]. The cause is unknown and it has been associated with different pathologic conditions: adverse drug reactions, autoimmune diseases, graft versus host disease, and cancer. These associations make the immune-mediated pathogenesis the most likely one. Histology is characterized by marked cholestasis without evidences of an accompanying hepatitis, but with a characteristic loss of bile ducts in the portal tracts. Clinical outcome is hepatic failure unless the underlying disease can be appropriately cured. We present a case of a VBDS that heralded the successive diagnosis of Hodgkin’s lymphoma (HL).

## Case presentation

**Figure 1:**
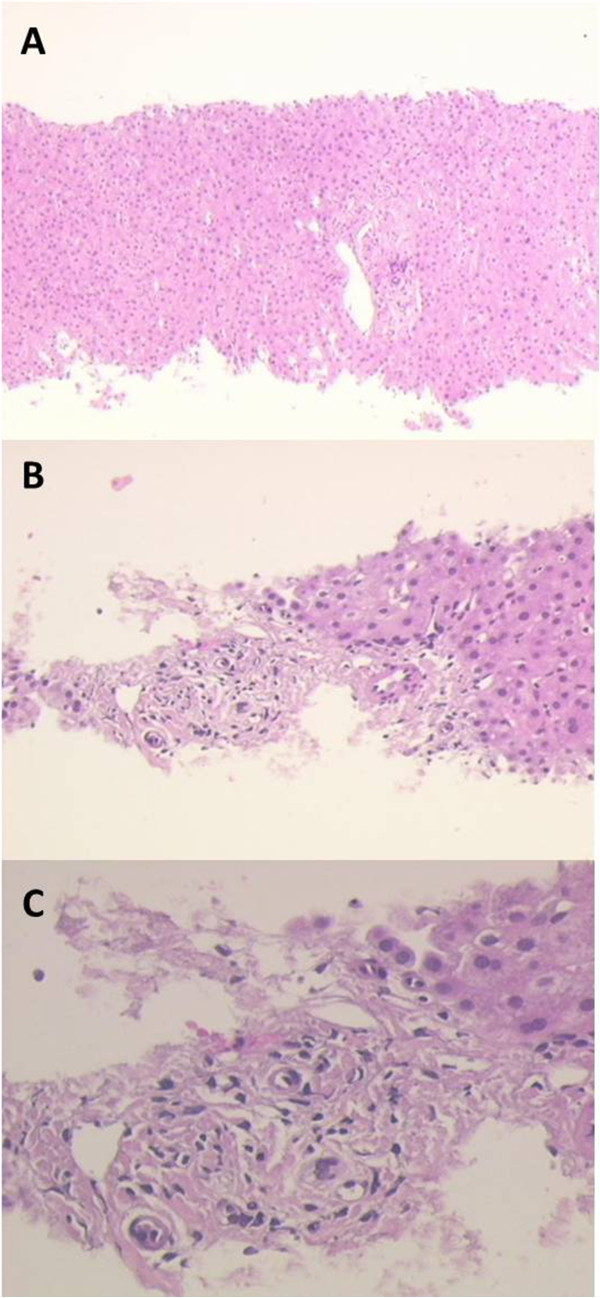
**Paraffin embedded section of the liver biopsy stained in hematoxylin-eosin showing fibrotic portal spaces without evidence of biliary interlobular ducts.** (Panel **A** 5x magnification, Panel **B** 20x magnification, Panel **C** 40x magnification).

A 42-year-old Caucasian woman was admitted to an infectious disease unit because of jaundice and fever. Her medical past history was unremarkable except for a mild allergy to cat hair. On admission her abnormal chemical tests were: total and direct bilirubin of 18.3 milligrams/deciliter (mg/dL) and 15.3 mg/dL, respectively (normal value up to 1.3 and 0.4 mg/dL, respectively); aspartate (AST) and alanine aminotransferase (ALT), 151 and 322, respectively (normal value up to 35 units/liter (U/L) and 40 U/L, respectively); alkaline phosphatase (ALP) 322 U/L (normal value up to 240 U/L); gamma-glutamyl transpeptidase (GGT) 482 U/L (normal value up to 35 U/L). Blood counts were normal except for leukocytosis (15170/μL). The patient was not taking any drug, and an extensive infectious disease work-up was negative. Particularly, hepatotropic viruses such as hepatitis A virus (HAV), hepatitis B virus (HBV) and hepatitis C virus (HCV) were excluded as well as Epstein-Barr virus (EBV) and cytomegalovirus (CMV). A computed tomography (CT) scan showed enlarged mediastinal lymph nodes without any abdominal pathological feature, and no sign of biliary tract dilatation. At that time, the patient was referred to our center, and a mediastinal biopsy was performed showing a picture of classic HL of nodular sclerosis histotype. Furthermore, the most common antibodies responsible for autoimmune hepatitis (ANA - antinuclear antibodies, ASMA – anti-smooth-muscle antibodies, anti-LKM1 – anti-liver-kidney microsomal type 1, anti-SLA - antibodies against soluble liver antigen, AMA - antimitochondrial antibody, antiphospholipid antibodies) were evaluated without any pathological findings. Since biochemical cholestasis had worsened (total bilirubin 33.8 mg/dL), a hepatic biopsy was performed showing marked cellular and canalicular perivenous cholestasis. Remarkably, bile ducts were absent (Figure 1) in all seven portal tracts examined and no sign of HL could be detected in a picture consistent with a VBDS [1]. Bone marrow trephine was negative. We also carried out a positron emission tomography (PET) scan that showed increased 18 F-fluoro-deoxy-glucose (18 F-FDG) uptake in multiple mediastinal lymph nodes defining a stage IIB HL. She was started on ursodeoxycholic acid (UDCA) (1350 mg daily) and prednisone (50 mg daily). A dramatic improvement of her clinical and biochemical conditions occurred with a decreased total bilirubin to 10 mg/dL. At this point, doxorubicin, bleomycin, vinblastine and dacarbazine (ABVD) chemotherapy was delivered and, after 4 cycles, we performed interim restaging with the PET scan showing a complete response. Therefore, she subsequently received two additional cycles of ABVD with a complete response confirmed after six cycles. Despite the fact that total bilirubin was increased at the time of the first cycle, we delivered a full dose of doxorubicin and vinblastine. At completion, prednisone was tapered and UDCA interrupted. The patient declined to receive a hepatic biopsy at the end of chemotherapy. Six years later, she is still in complete remission with a thoroughly normal hepatic function.

## Conclusion

Several conditions may cause jaundice in HL. Biliary obstruction secondary to lymph nodes enlargement, hemolysis, hepatotropic and other viruses (e.g. CMV) are among the most common causes. Hepatic direct involvement by HL is a relatively rare, but well known complication [2]. The association of cholestatic jaundice and hepatic bile duct loss (a picture called “ductopenia”) in HL has been described in other 39 other cases in the literature (Table 1). This is a severe complication with a high rate of death in a neoplastic disease otherwise curable in more than 80% of patients. In 18 HL patients steroid plus lymphoma treatment (both chemotherapy and radiotherapy) was described to improve hepatic function and eventually led to complete remission of both hepatic and Hodgkin’s diseases. All other cases died because of hepatic failure. A clear pathogenetic relationship between HL and VBDS cannot be identified. Nevertheless, the concomitance of the two diseases and the case reported by Allory [3] makes the hypothesis of VBDS likely to be a paraneoplastic manifestation of HL. Furthermore, Bruguera [4] has shown hepatic sinusoidal dilatation (a feature of VBDS) in 90% of patients presenting systemic symptoms (so called B symptoms) without any evidence of direct liver involvement by the lymphoma. This datum suggests the role of HL as the trigger event of cytokine-mediated hepatic damage [5]. There is evidence that biliary epithelial cells [6] express major histocompatibility complex (MHC) antigens (both class I and II) as well as adhesion molecules like intercellular adhesion molecule 1 (ICAM-1) in response to cytokines produced by HL. In this context, both types of molecules may contribute to adhesion and cytotoxicity due to T lymphocytes. As of today, there is no recognized treatment for VBDS. We, as well as other authors, used UDCA because of its demonstrated activity in primary biliary cirrhosis. Indeed, beneficial effects of treatment with UDCA have been reported in other immune-mediated disease involving the biliary tree as primary sclerosing cholangitis [7] or hepatic involvement by graft versus host disease [8]. UDCA acts at three levels: increasing the expression of transporter proteins into the canalicular membrane, inhibiting apoptosis induced by toxic bile acids, and altering the composition of the micelles of bile acids [7].

**Table 1:** **Summary of all cases reported in the literature regarding vanishing bile duct syndrome in lymphomas**

Author	Number of patients	Hodgkin lymphoma treatment*	Dosage	Ursodeoxycholic acid	Outcome
Bouroncle B. [9]	2	Yes	Full	Unknown	Hepatic failure
		Yes	Reduced		Hepatic failure
Juniper K. [10]	1	Yes	Reduced	Unknown	Hepatic failure
Groth C. [11]	1	Yes	Full	Unknown	Hepatic failure
Perera D.R. [12]	3	Yes	Full	Unknown	Hepatic failure
		Yes	Full	Unknown	Cure
		Yes	Full	Unknown	Cure
Piken E.P. [13]	1	Yes	Full	Unknown	Unknown
Trewby P.N. [14]	4	Yes	Full	Unknown	Cure
		Yes	Full	Unknown	Cure
		Yes	Full	Unknown	Unknown
		No	No	Unknown	Unknown
Lieberman D.A. [15]	1	No	No	No	Respiratory arrest
Birrer M.J. [16]	1	Yes	Full	Unknown	Sepsis
Hubscher S.G. [1]	3	Yes	Reduced	No	Hepatic failure
Jansen P.L.M. [17]	1	Yes	Full	Late	Hepatic failure
Warner A.S. [18]	1	Yes	Full	No	Cure
Gottrand F. [19]	1	No	No	No	Hepatic failure
Crosbie O.M. [20]	1	Yes	Full	Yes	Cure
De Medeiros B.C. [21]	2	Yes	Full	No	Hepatic failure
			Full		
Yalcin S. [22]	2	No	No	No	Hepatic failure
		Yes	Full	No	Cure
Dourakis S.P. [23]	1	Yes	Reduced	No	Hepatic failure
Yusuf M.A. [24]	1	No	No	Late	Hepatic failure
Rossini M.S. [25]	1	Yes	Full	No	Hepatic failure
Allory Y. [3]	1	Yes	Full	Yes	Cure
Ozkan A. [26]	1	No	No	Late	Hepatic failure
Ripoll C. [27]	2	Yes	Full	No	Hepatic failure
		Yes	Full	Yes	Cure
Komurcu S. [28]	1	Yes	Full	No	Hepatic failure
Liangpunsakul S. [29]	1	Yes	Full	No	Cure
Guliter S. [30]	1	Yes	Full	Late	Hepatic failure
Córdoba Iturriagagoitia A. [31]	1	Yes	Full	No	Cure
Han W.S. [32]	1	No	No	No	Recurrent Hodgkin’s Lymphoma
Schmitt A. [33]	1	No	No	No	Hepatic failure
Barta S.K. [34]	1	Yes	Full	No	Cure
DeBenedet A.T. [35]	1	Yes	Reduced	No	Hepatic failure
Leeuwenburgh I. [36]	1	Yes	Reduced	No	Cure
Pass A.K. [37]	2	Yes	Reduced	Yes	Cure
		Yes	Reduced	No	Hepatic failure
Ballonoff A. [38]	1	Yes	Full	No	Cure
Umit H. [39]	1	Unknown	Unknown	Unknown	Unknown
Gill R.M. [40]	1	Yes	Reduced	No	Hepatic failure
Foramiti S. [41]	1	Yes	Full	Yes	Hepatic failure
Gagnon MF [42]	1	Yes	Full	No	Cure
Wong KM [43]	1	Yes	Full	No	Cure
Aleem A1 [44]	1	Yes	Full	No	Hepatic failure
Nader K [45]	1	Yes	Full	No	Hepatic failure
Our patient	1	Yes	Full	Yes	Cure

*either chemotherapy or radiotherapy.

In the present report we stress two aspects. First, the therapeutic choice to use high-dose of UDCA [46] and prednisone which rapidly determined an improvement of our patients. Secondly, although still icteric, we delivered a full dose chemotherapy achieving a sustained complete remission that, in a paraneoplastic disease, is the most important result. An appropriate aggressive treatment (either chemotherapy or radiotherapy) was reported in all patients who were, in the end, cured (Table 1). Notwithstanding, several good reasons stand for a dose reduction in both doxorubicin and vinblastine in a jaundiced patient, we emphasize the “full dose” chemotherapy choice. ABVD chemotherapy is conceived as a 2-weekly treatment and, therefore, 25 mg/m^2^ doxorubicin was felt appropriate regardless of jaundice. Indeed, it is half the dose used in other combinations (*e.g.* CHOP regimen - Cyclophosphamide, Doxorubicin, Vincristine, Prednisone or CODOX-M/IVAC – Cyclophosphamide, Vincristine, Doxorubicin, Methotrexate, Ifosfamide, Etoposide, Cytarabine [47, 48]). Moreover, it is well known that the ABVD dose intensity affects the HL outcome [49, 50]. Lastly, only 1 out of 8 (12%) patients receiving an upfront reduced chemotherapy overcame the vanishing bile duct, compared to 51% (17/33) of patients treated with upfront full dose chemotherapy (see Table 1). In these patients the efficacious treatment of the lymphoma preceded the hepatic improvement and, ultimately, the cure.

This case report demonstrates that in the presence of a histological diagnosis of HL associated with signs of cholestasis and liver function impairment without gross liver involvement or biliary tract dilatation, the prompt treatment of the underlying disease in association with high doses of UDCA is crucial. Indeed, data available in the literature strengthen the theory that the therapy able to restore liver function is full dose chemotherapy only. In this setting, waiting for the histological confirmation of VBDS may be harmful because a delayed start might be detrimental to patient outcome. Indeed, liver impairment could evolve to liver insufficiency and eventually to death as is clearly outlined in Table 1.

In conclusion, VBDS must be considered a rare but possible complication of HL. Its prompt recognition and appropriate treatment can dramatically affect patients’ outcome.

## Consent

Written informed consent was obtained from the patient for publication of this Case Report and any accompanying images. A copy of the written consent is available for review by the Editor-in-Chief of this journal.
